# High coercivity, anisotropic, heavy rare earth-free Nd-Fe-B by Flash Spark Plasma Sintering

**DOI:** 10.1038/s41598-017-11660-9

**Published:** 2017-09-11

**Authors:** Elinor Castle, Richard Sheridan, Wei Zhou, Salvatore Grasso, Allan Walton, Michael J. Reece

**Affiliations:** 1 Queen Mary, University of London, Mile End Road, London, E1 4NS UK; 2 University of Birmingham, Edgbaston, Birmingham, West Midlands B15 2TT UK

## Abstract

In the drive to reduce the critical Heavy Rare Earth (HRE) content of magnets for green technologies, HRE-free Nd-Fe-B has become an attractive option. HRE is added to Nd-Fe-B to enhance the high temperature performance of the magnets. To produce similar high temperature properties without HRE, a crystallographically textured nanoscale grain structure is ideal; and this conventionally requires expensive “die upset” processing routes. Here, a Flash Spark Plasma Sintering (FSPS) process has been applied to a Dy-free Nd_30.0_Fe_61.8_Co_5.8_Ga_0.6_Al_0.1_B_0.9_ melt spun powder (MQU-F, neo Magnequench). Rapid sinter-forging of a green compact to near theoretical density was achieved during the 10 s process, and therefore represents a quick and efficient means of producing die-upset Nd-Fe-B material. The microstructure of the FSPS samples was investigated by SEM and TEM imaging, and the observations were used to guide the optimisation of the process. The most optimal sample is compared directly to commercially die-upset forged (MQIII-F) material made from the same MQU-F powder. It is shown that the grain size of the FSPS material is halved in comparison to the MQIII-F material, leading to a 14% increase in coercivity (1438 kA m^−1^) and matched remanence (1.16 T) giving a BH_max_ of 230 kJ m^−3^.

## Introduction

In the drive to reduce our dependence upon fossil fuels and to curb fuel emissions, the use of green technologies such as electric vehicles, wind turbines and electric generators is rapidly growing. The success of the green technologies revolution relies heavily on the development of suitable and economically viable, high performance hard magnetic materials. Currently, the best performing hard magnets are the rare-earth transition-metal (RE-TM) Nd-Fe-B based materials, which have produced the highest recorded maximum energy product (BH_max_ = 474 kJ m^−3^)^1^. The outstanding performance of these magnets is derived from the main hard magnetic Nd_2_Fe_14_B phase, which has a good saturation magnetisation, M_S_, due to its relatively high Fe content and which exhibits a high coercivity, H_c_, due to the high degree of magnetocrystalline anisotropy introduced by the RE elements. However, one significant drawback to the Nd_2_Fe_14_B intermetallic compound is its negative temperature coefficient of coercivity (TCC); which leads to a substantial irreversible loss of H_c_ and remanence, B_r_, at elevated temperatures. This can be severely detrimental to the performance of green technologies such as electric motors, in which temperatures can reach upwards of 423 K.

In order to improve the magnetic performance of Nd-Fe-B at elevated temperatures, up to 10 at.% of the heavy rare earth (HRE) element Dy is added, which substitutes some of the Nd in the Nd_2_Fe_14_B phase; increasing its magnetocrystalline anisotropy and therefore H_c_. While this strategy has been successful in terms of improving the high temperature performance of the magnets, the antiparallel coupling of the Dy magnetic moments with the Fe moments leads to a decrease in B_r_ and, subsequently, BH_max_. Additionally, and perhaps more critically, the inclusion of a large proportion of the critical HRE introduces a price and supply instability/risk for all non-Chinese Nd-Fe-B production^2^.

In order to substantially reduce the HRE content, one successful strategy has been to concentrate the HRE at the surface of the Nd_2_Fe_14_B grains to create a core-shell type structure; with the shell being enriched with HRE. Since the nucleation and growth of reverse domains most often occurs at defects on the surfaces of the grains, this ensures that the HRE is concentrated only where a higher coercivity is most needed; and avoids unnecessary reduction of the B_r_ in the bulk of the grains. This has typically been achieved using various forms of a grain boundary diffusion process (GBDP), in which the surface of a sintered magnet is coated in the pure HRE (or its oxide or fluoride) followed by high temperature heat treatment (~1073 K) and low temperature ageing. This enables the HRE to diffuse along the grain boundaries and into the Nd_2_Fe_14_B grains to form the core-shell structure^3–9^.

Considerable research is also being undertaken to develop promising new materials that are completely free of critical RE’s, for example: ordered L1_0_ -FeNi^10–13^, α“-Fe_16_N_2_^14,15^, exchange-spring type hard-soft nanocomposites^16,17^, Heusler compounds^18^ and ThMn_12_-type compounds^19^. However, no RE-free material has yet been able to exceed or even match the combined performance and processability of bulk Nd-Fe-B. Most RE-free solutions present substantial challenges in respect to their synthesis and processing, since their magnetic performance is often linked to the creation and maintenance of low temperature phases, metastable phases and nanoscale microstructures. Consolidating such materials to full density, without at least a partial loss of the hard magnetic properties or without diluting the properties through the use of a resin material, is therefore a challenging task.

The development of high performance HRE-free Nd-Fe-B materials has become a more economically and technologically attractive option. To increase the coercivity of Nd_2_Fe_14_B without using HREs, the focus shifts to the extrinsic factors affecting coercivity; where microstructural optimisation (refined grain size, shape, aspect ratio, composition and character of the grain boundary phase, etc…) allows a greater proportion of the intrinsic magnetic properties to be transferred to the final magnet. Brown^20,21^ showed that coercivity should be equal to or greater than the anisotropy field, H_A_ = 2K_1_/M_S_, where K_1_ is the first magnetic anisotropy constant. In practice, this systematically overestimates H_c_ by up to one order of magnitude; leading to what is known as Brown’s paradox. This shortfall in coercivity can be explained through the existence of defects in the material, which act as preferential nucleation sites for magnetisation reversal at much lower coercive fields. In conventional sintered Nd-Fe-B, magnetisation reversal takes place via domain wall motion following the nucleation of a reverse domain at a defect site. Domain wall motion is energetically less demanding in this case, since the micron-sized grains can comfortably accommodate several domain walls. If the size of the grains is decreased to the single-domain size, which for Nd-Fe-B is around 300 nm, then the nucleation and growth of a reverse domain is energetically more difficult than for reversal in a multi-domained large grain; hence the coercivity is enhanced^20^.

Further increases in the BH_max_ of the material can be obtained by increasing the remanence through texturing. A crystallographic alignment of the ‘easy’ axis of magnetisation (the crystallographic ‘c’ axis in the case of Nd_2_Fe_14_B) maximises the achievable B_r_ when the material is magnetised along this direction. Since BH_max_ increases quadratically with respect to the remanence, the introduction of magnetic anisotropy therefore leads to substantial increases in the energy product. The ideal, HRE-free high performance Nd-Fe-B magnet would therefore consist of crystallographically aligned, single domain sized grains.

Conventional methods for the production of anisotropic (high remanence), nanoscale (high coercivity) Nd-Fe-B-based magnetic materials involve multiple processing steps and typically employ high temperatures, pressures and long processing times; leading to production costs that are several times that of the raw materials costs. For example, where anisotropic starting materials (such as Hydrogenation Disproportionation Desorption Recombination, HDDR, powder^22–24^ or single domain size particles^25^) are employed, magnetic pulse alignment and compaction can be used to produce anisotropic green parts; however, long sintering (1–2 hours at ~1000 °C) and annealing (1–2 hours at ~600 °C) treatments are then required in order to produce high density materials. Excellent magnetic alignment can be achieved using this route, leading to B_r_ values upwards of 1.4 T and BH_max_ values greater than 400 kJ m^−3^. However, H_c_ values rarely exceed ~800 kA m^−1^, which is largely due to the excessive growth of the main hard magnetic Nd_2_Fe_14_B grains to around 5–10 μm. As such, these types of anisotropic powders are more commonly consolidated at low temperatures using resin bonding techniques. In this case the introduction of a non-magnetic resin significantly reduces the BH_max_.

Alternatively, to obtain a finer grain size and therefore higher coercivity in the absence of Dy, rapid solidification melt spun ribbons may also be employed as the starting materials. Since these powders are magnetically isotropic (consisting of multiple, randomly oriented Nd_2_Fe_14_B grains), they cannot be formed into an aligned green compact. Instead, a three-step process is employed to induce texture into the isotropic starting material, involving: cold compaction to green density; isotropic hot pressing to full density; and then a final die upset or backward extrusion step^26^. The forging step (die-upset or backward extrusion) leads to anisotropic grain growth into crystallographically aligned plate-like grains through a stress-enhanced solution-precipitation mechanism^27^, subsequently producing a desirable fine grained and magnetically anisotropic material. However, processing costs typically triple the cost of the raw material and hence this type of material only becomes financially feasible to produce when Dy prices soar.

In recent years, progress in Electric Current Assisted Sintering techniques (ECAS) has led to the development of fully dense, fine-grained Nd-Fe-B materials^28–30^. Such techniques employ high pressures and high heating rates; brought about through the application of large electric currents to the highly electrically conductive graphite die sets. This leads to a rapid Joule heating of the die (and powder, if it is electrically conductive) and therefore a rapid sintering of the material to full density in a matter of minutes. The rapid nature of the technique means that Nd_2_Fe_14_B grain growth can be limited to sub-micron sizes, increasing the coercivity. However, in order to obtain anisotropic materials with high energy products, additional die-upset post-processing steps are still required^31,32^.

We previously reported the results of an initial investigation into the use of a novel ‘Flash Spark Plasma Sintering’ (FSPS) technique^33^ for the production of anisotropic, nanograined Nd-Fe-B materials (MQU-G powder containing Dy)^34^. In this work, a Spark Plasma Sintering (SPS) furnace was configured to perform Flash Sintering (FS) on melt-spun Nd-Fe-B containing 3.72 wt.% Dy. The materials produced exhibited high degrees of anisotropy, high coercivities (>1600 kA m^−1^) and nanoscale microstructures; demonstrating the potential benefits of this technique. FS is a recently discovered phenomenon which has generated much interest in the ceramics community. Here, extremely rapid sintering (in seconds as opposed to minutes or hours) to near full density is brought about due to a sudden increase in electrical conductivity and diffusion, which occurs beyond a critical combination of sample temperature and applied voltage gradient^35^. FSPS is possible only in materials which have sufficient electrical conductivity, since the SPS voltage output is commonly limited to around 10 V. In the FSPS configuration, a green compact is placed between two graphite punches, with graphite felt surrounding the sample in order to achieve pre-heating (if required) and provide thermal insulation. In the absence of a graphite die, the majority of the current is forced through the sample to produce flash sintering, while at the same time sample deformation occurs. As such, the FSPS technique produces a simultaneous densification and die-upsetting of the sample; and, due to its extremely rapid nature, helps to retain a nanoscale microstructure at the same time as producing crystallographic texture in the material.

In the present work, the results of a more extensive study into the FSPS processing of Dy-free melt-spun Nd-Fe-B is reported. Through multiscale microstructural and physical properties characterisation, the FSPS process has been analysed and optimised to produce fully dense, anisotropic materials in 10 s from green compacts. It is shown, through direct comparison with a commercial ‘die upset’ material fabricated from the same starting powder (MQIII-F, supplied by neo Magnequench), that the magnetic properties of the optimised materials possess enhanced coercivities and matching remanence; thereby exceeding the current state-of-the-art.

## Materials and Methods

The hard magnetic Nd-Fe-B powder utilised in this work was a commercial, Dy-free melt spun material (“MQU-F”) with the composition Nd_30.0_Fe_61.8_Co_5.8_Ga_0.6_Al_0.1_B_0.9_ (wt.%, minor additions not included) supplied by neo Magnequench. These magnetically isotropic ribbons consist of randomly oriented equiaxed grains which are typically 20–50 nm in diameter. The microstructure of these ribbons is given in Supplementary Figure A. The powder has a specified remanence of 0.81 T, coercivity of 754 kA m^−1^ and BH_max_ of 102 kJ m^−3^.

In addition, neo Magnequench supplied an anisotropic die-upset forged block of material, fabricated from the same batch of MQU-F powder using the conventional three step die-upset forging route^27^. This was used as a benchmark material for direct comparisons with the materials produced via FSPS. This material exhibited a remanence of 1.21 T, coercivity of 1250 kA m^−1^ and BH_max_ of 260 kJ m^−3^ (not corrected for self-demagnetisation), which is used as the baseline for comparison with samples produced in this paper.

For preparation of samples using FSPS, ~70% green density compacts were first prepared by SPS (FCT HPD 25; FCT Systeme GmbH, Rauenstein, Germany). For each sample a Ø = 20 mm graphite die, lined with graphite foil, was charged with 20 g of the MQU-F powder. This was then placed into the SPS furnace and sintered under a 5 Pa vacuum and 50 MPa pressure, for 30 s at 550 °C, using a heating rate of 100 °C min^−1^. This produced a Ø ≈ 20 mm and H ≈ 12 mm green compact. Cold-pressing could also have been used to make the green compact, as demonstrated by McKinnon *et al*.^36^. The only pre-requisite is that the compact should be robust enough to withstand the minimum 5 kN pressure applied by the SPS pistons. For the FSPS step, a green compact was placed into the SPS furnace, between two Ø = 40 mm graphite punches and wrapped in graphite felt. The SPS program was then set to heat to 450 °C (the minimum temperature at which the pyrometer, focused onto the inside of the top punch at a distance of 4 mm from the upper surface of the sample, can take an accurate reading), followed by a dwell of 1 minute at this temperature in order to evenly preheat the sample. Samples were then subjected to a 10 s pulse of continuous direct current, by discharging under a set power defined as a percentage of the maximum power capacity of the machine. The actual heating power applied to the samples, along with measured temperature, shrinkage rates, current and voltage uses, applied force, vacuum pressure etc… could then be obtained from the SPS data log collected during processing. Based on the fact that the temperature profiles recorded by the pyrometer during FSPS were similar to those recorded during the trials reported in the previous study^34^ (where direct temperature measurement of the sample was made through the insertion of a thermocouple into the centre of the sample), the heating rates achieved are estimated to be up to 2660 K s^−1^. Typical power, deformation rate and temperature outputs for the 10 s flash process are shown in Supplementary Figure B. Initially, five samples were produced in this way using different heating powers and while applying only the minimum 5 kN pressure, under a 5 Pa vacuum; as in our previous work^34^. After the 10 s FS step, the sample was left to cool in contact with the water cooled pistons (approximately 200 K min^−1^).

Magnetic measurements were performed in the ‘easy’ and ‘hard’ directions of magnetisation on 4 × 4 × 4 mm cubes cut from the centre of the FSPS and MQIII-F samples using a LakeShore 7300 vibrating sample magnetometer (VSM) at room temperature.

Sample densities were measured using Archimedes method in water. Measurements were performed on 1 cm^2^ pieces cut from the centre of the sintered samples (typically 5–6 mm thick), since the edges of the samples experienced greater heat loss during FSPS and therefore exhibited excess porosity.

Scanning electron microscopy was performed in secondary electron imaging (SEI) mode on sample fracture surfaces using an FEI Inspect™-F field emission gun scanning electron microscope (FEG-SEM). A working distance of 10 mm, spot size 3.5 and accelerating voltage of 20 kV were employed. Transition electron microscopy (TEM) was performed using a Jeol 2100 high resolution TEM in bright field (BF) image mode at 200KV on samples prepared using a FEI Quanta 3D FEG FIB (focused ion beam) SEM.

### Data availability statement

The datasets generated during and/or analysed during the current study are available from the corresponding author on reasonable request.

## Results and Discussion

**Figure 1:**
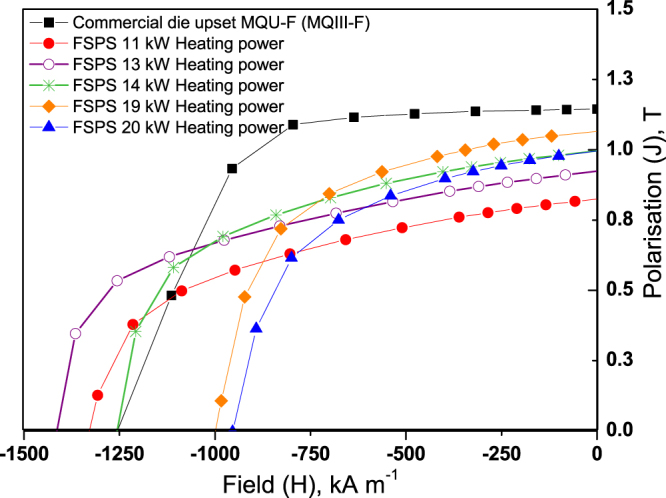
Demagnetisation curves of the first FSPS samples, produced by discharging under different heating powers, in comparison to the commercial MQIII-F material measured using the same method.

**Figure 2:**
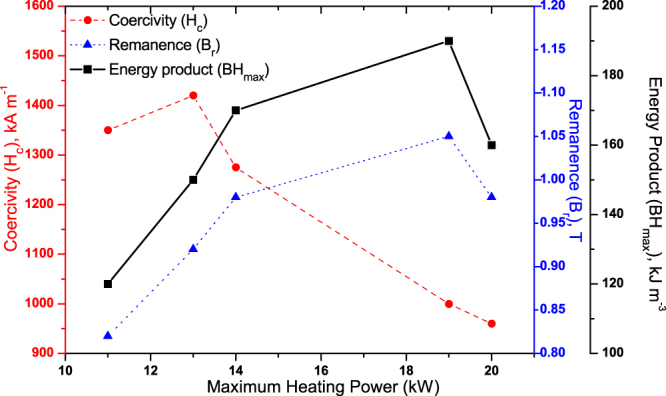
Summary of the measured coercivity, remanence and BH_max_ of the initial FSPS samples plotted as a function of maximum heating power.

Each of the five FSPS samples produced initially by discharging under different heating powers was magnetically characterised using a VSM in the ‘easy’ and ‘hard’ directions of magnetisation. The demagnetisation curves of each of these samples in the ‘easy’ direction of magnetisation are plotted in Fig. 1, along with the data for the commercial die upset MQIII-F material for direct comparison. Figure 2 summarises the measured H_c_, B_r_ and BH_max_ of each of the FSPS samples as a function of the maximum recorded heating powers.

**Figure 3:**
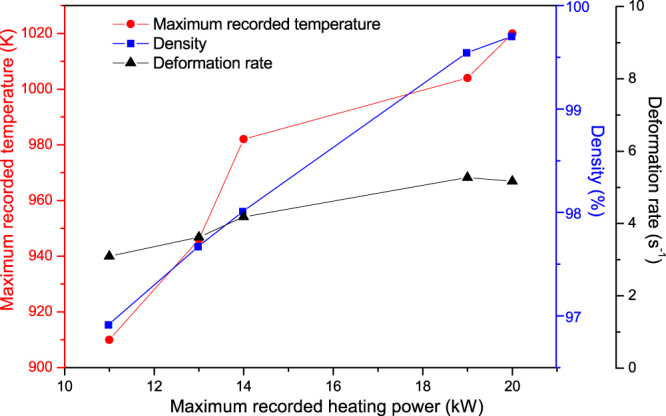
Deformation rate, maximum recorded temperature and density as a function of maximum recorded heating power for the five initial FSPS samples. Note: the maximum recorded temperature is measured from the inside of the top punch, 4 mm from the top surface of the sample and hence will be a significant underestimation of the actual sample temperature; it is intended only as a relative guide.

All of the initial samples produced by FSPS exhibit a lower remanence than the commercial MQIII magnet, as well as a reduced loop squareness (Fig. 1); indicating that the degree of crystallographic alignment achieved in the FSPS samples is not optimal. The highest B_r_ values were achieved in the samples discharged under the highest heating powers. This can be attributed to the higher temperatures reached under these powers and the subsequent higher deformation rates (Fig. 3); both of which increase the final density of the samples and boost the stress-enhanced solution-precipitation mechanism responsible for plate-like grain growth and alignment^27^. This mechanism for plate-like grain growth relies upon the melting of the Nd-rich grain boundary phase and the dissolution of the Nd_2_Fe_14_B grains into the liquid phase. The degree of Nd_2_Fe_14_B dissolution depends upon the orientation of the grains with respect to the pressing direction, since the Nd_2_Fe_14_B compound has anisotropic mechanical properties. Along the c-axis, the elastic constant is smaller than along the a-axis and hence the strain energy in grains stressed parallel to their a-axis is higher. These ‘unfavourably’ oriented grains therefore dissolve at a higher rate than the ‘favourably’ oriented grains (with their c-axis lying within ±15° to the stress direction), setting up concentration gradients in the liquid phase. This provides the driving force for diffusion and the growth of the ‘favourably’ oriented grains at the expense of the ‘unfavourably’ oriented grains; occurring in a plate-like manner in order to maximise the lowest surface energy {001} planes. Subsequently, the plates grow preferentially along the a-axis, naturally leading to an alignment of the plates perpendicular to the pressing direction. This is additionally facilitated by grain boundary sliding under the applied pressure, resulting in a crystallographically aligned dense material. Li and Graham^27^ noted that, while sufficient diffusion rates are necessary for the activation of this grain growth mechanism, relatively high diffusion rates will lead to a removal of the concentration gradients; i.e. the process depends on the relative rates of diffusion and dissolution. A relatively high diffusion rate will therefore remove the driving force for orientation-selective grain growth, leading to a decrease in the degree of texturing; and, since atomic attachment kinetics no longer limit the crystal growth process, the equiaxed (rather than plate-like) coarsening of grains will take place via conventional Ostwald ripening. This can account for the relative drop in B_r_ (and H_c_) seen in the FSPS sample sintered under the highest applied heating powers (Fig. 2).

**Figure 4:**
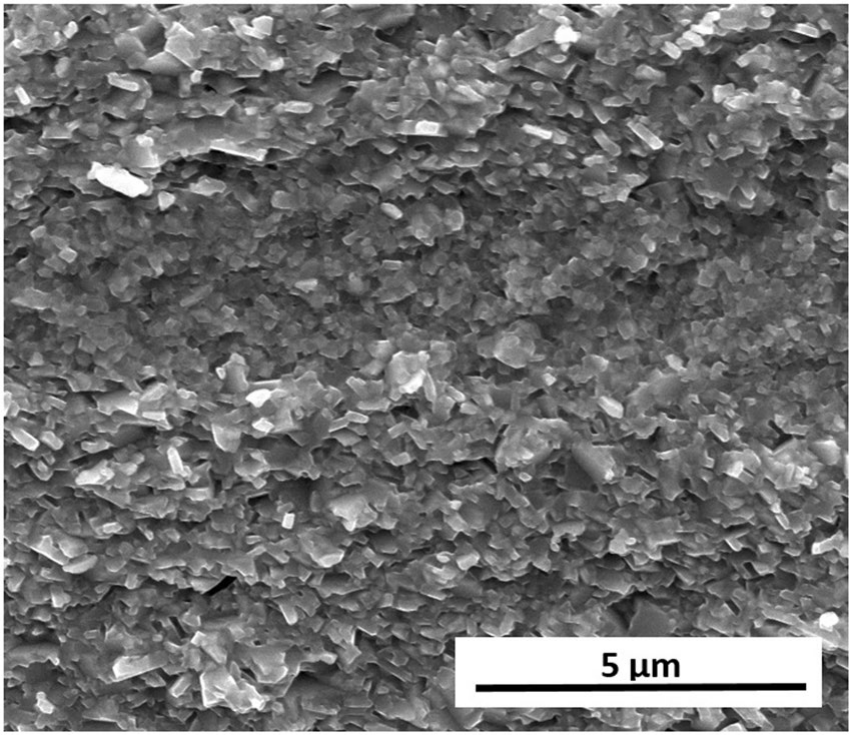
Typical microstructure of the sample processed under the lowest heating power of 11 kW under a constant 5 kN force, showing minimal grain growth and crystallographic alignment.

In contrast to the FSPS samples processed under the highest heating powers, the coercivity of the samples processed under the lowest heating powers exceeded that of the commercial MQIII-F material (Fig. 3). The short processing time and lower maximum processing temperatures resulted in minimal grain growth, thereby retaining single domain-size grains in the final sintered product (higher coercivity) with only a small degree of anisotropic grain growth and reduced crystallographic alignment (lower remanence) (Fig. 4).

**Figure 5:**
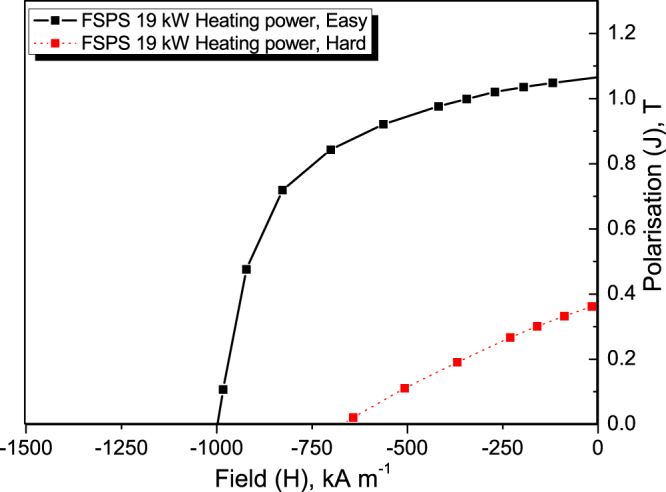
Magnetic anisotropy (demagnetisation curves measured in the ‘easy’ and ‘hard’ directions of magnetisation) of the best initial sample.

It can be observed from Fig. 2 that the highest BH_max_ was achieved using a heating power of 19 kW; which coincides with the highest remanence (1.05 T), yet (as noted previously) a significant drop in coercivity to below 1000 kA m^−1^. The demagnetisation loops in the ‘easy’ and ‘hard’ directions of magnetisation for this sample are shown in Fig. 5. Significant anisotropy is evident between the ‘easy’ and ‘hard’ directions; with the magnetic anisotropy calculated to be 66% using equation (1). It should be noted that it was not possible to fully saturate the sample in the ‘hard’ direction of magnetisation, hence the appearance of a lower coercivity value in this orientation.

1 $$Magnetic\,Anisotropy=\frac{({B}_{r\coprod }-{B}_{r\perp })}{{B}_{r\coprod }}\times 100=66 \% $$

*Where B*_*R***||**_ = *Remanence parallel to the c-axis and B*_R⊥_ = *Remanence perpendicular to the c-axis*.

While the 19 kW sample is promising and offers the best magnetic properties out of the FSPS samples, there is still a considerable shortfall in coercivity and remanence in comparison to the commercial MQIII-F material. In order to understand the origin of this shortfall, the microstructures of the 19 kW sample and the MQIII-F material were investigated and compared using SEM and TEM imaging.

**Figure 6:**
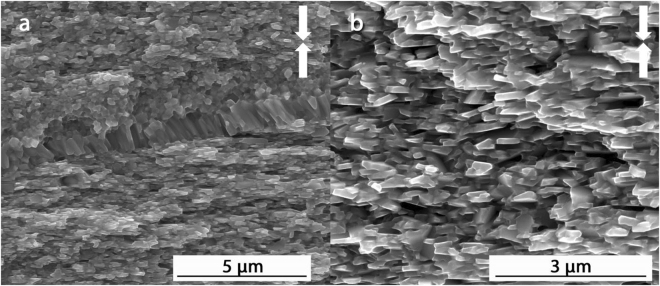
Typical SEM secondary electron images of the fracture surface of the commercial MQIII-F material. Arrows indicate pressing direction.

Figure 6 shows typical microstructures observed by SEM imaging of a fracture surface parallel to the pressing direction in the block of MQIII-F material. Throughout the majority of the block, the plate-like grains are relatively uniform in size, shape and orientation. The average measured length of the grains is 451 ± 131 nm and their average width is 122 ± 38 nm. In some regions there remain some small, equiaxed grains and some strings of columnar grains observed at the former interface between powder flakes, lying parallel to the pressing direction (Fig. 6, left image). This is an artefact common to materials made from melt spun ribbons^37^.

**Figure 7:**
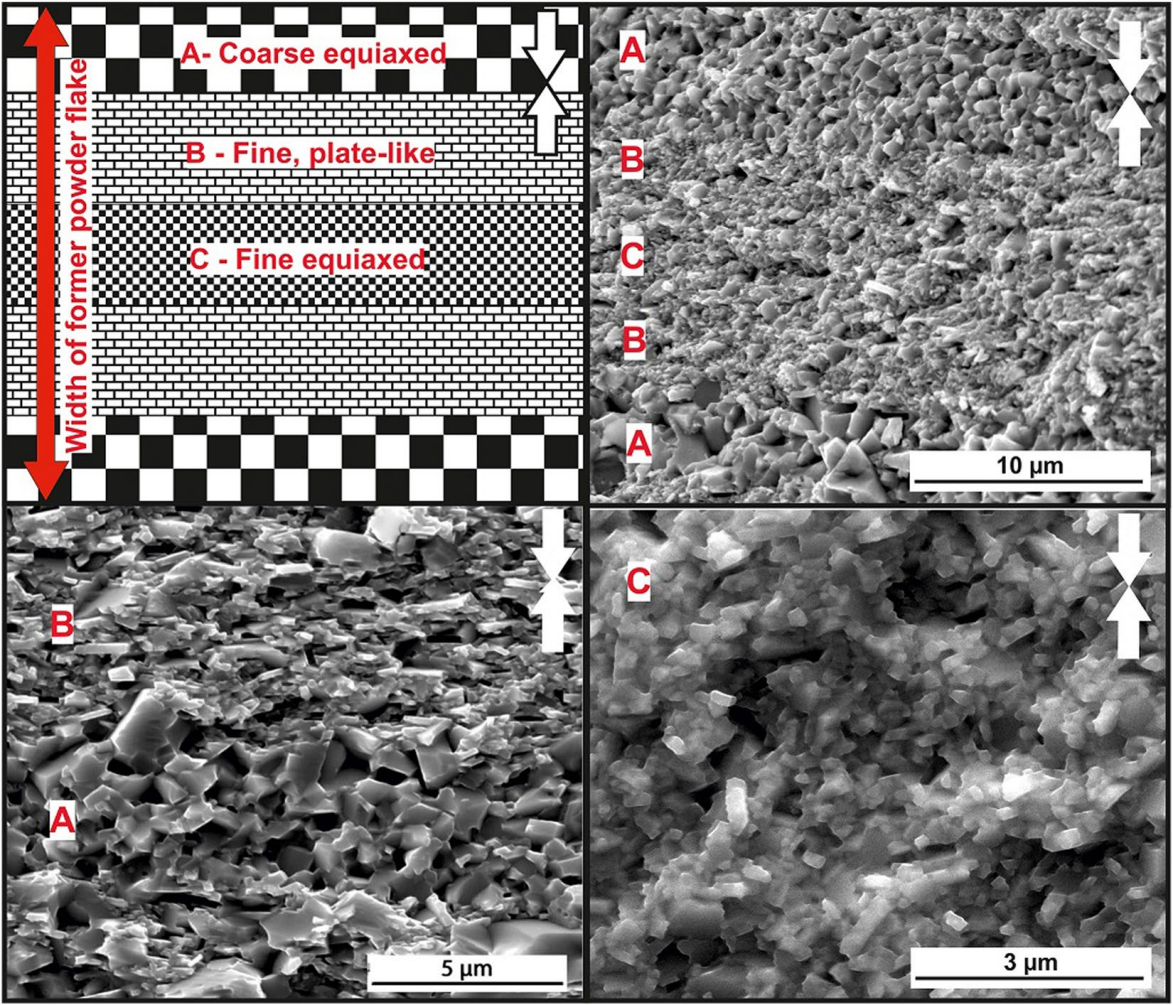
(Top left) diagram illustrating the typical microstructure observed at low magnification across the full width of a MQU-F powder flake, showing coarse equiaxed (**A**), plate-like aligned (**B**) and fine equiaxed (**C**) layers in the best initial FSPS sample (19 kW). The rest of the images are SEM secondary electron images taken from the fracture surface of the 19 kW FSPS sample, showing typical images of the different layers. White arrows indicate the pressing direction.

In comparison, the 19 kW FSPS sample displays a much coarser and more inhomogeneous microstructure (Fig. 7). While a significant proportion of the microstructure consists of aligned plate-like grains (B), large equiaxed grains (A) are also observed at the surfaces between the flake-like powder particles, and fine equiaxed grains (C) remain in the centre of the former flakes. This generally leads to a layered structure within the former flakes, as depicted in Fig. 7 (top left) and shown in the representative SEM images in the rest of Fig. 7. The coexistence of these different microstructures over the ~30 µm thickness of the powder flakes indicates the existence of steep temperature gradients during processing. These temperature gradients appear to have been localised at the scale of the powder particles. At the macro scale, the layered structure was observed across the full width of the sample; with very little variation between the centre and the edge.

The existence of coarse grains at the former powder flake surfaces and fine grains remaining at the centre of the flakes indicates that localised overheating occurred at the powder interfaces, leading to a decreasing temperature gradient into the flakes. There will consequently be a gradient in the dissolution and diffusion rates of Nd_2_Fe_14_B in the liquid grain boundary phase. At the particle interface, high temperatures lead to high diffusion rates; which removes the main driving force for orientation-based preferential grain growth (as discussed previously) and leads instead to equiaxed grain coarsening via Ostwald ripening. In the centre of the former powder flakes, the temperature is not sufficient to allow the adequate dissolution and diffusion of Nd_2_Fe_14_B during the 10 s FS step and hence there is very little grain growth and alignment. Naturally, there then exists a region between the interface and the centre of the flakes where conditions are ideal for plate-like grain growth and alignment; resulting in the observed layered structure.

**Figure 8:**
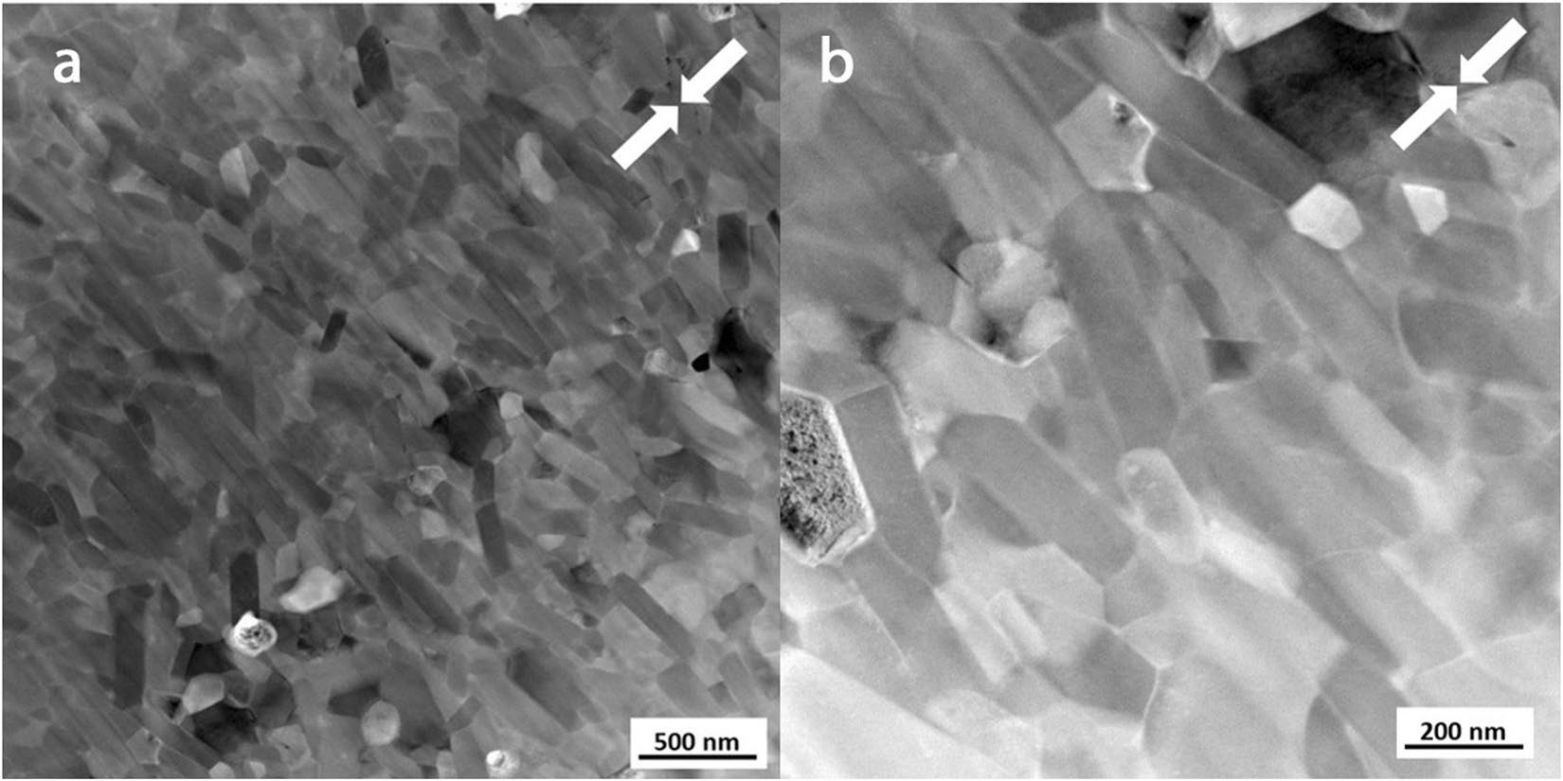
TEM images of the commercial die-upset MQIII-F material.

**Figure 9:**
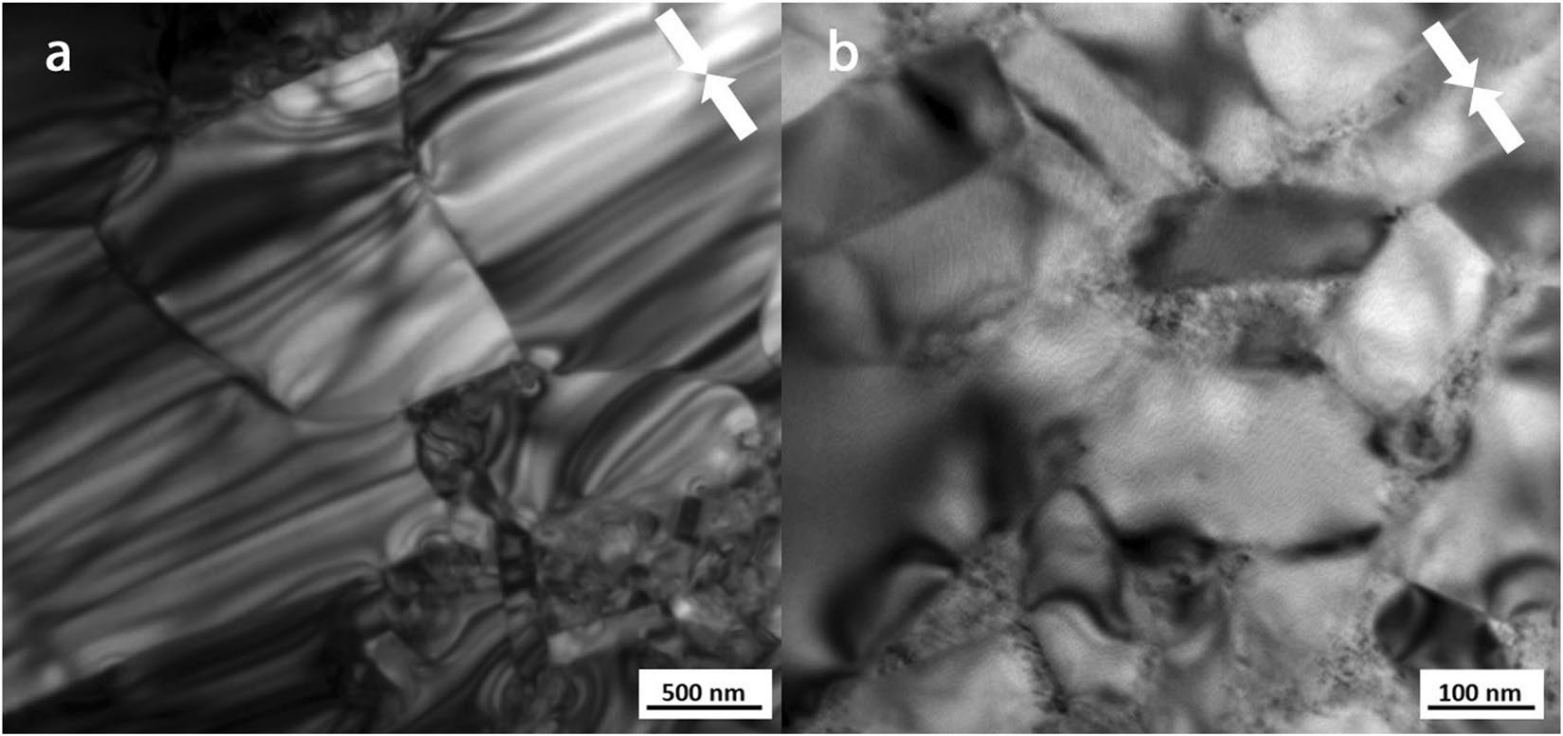
TEM images of the ‘best’ initial FSPS MQU-F sample (19 kW, 10 s, 5 kN). (**a**) Large equiaxed grains at particle-particle interface. (**b**) Smaller plate-like grains in the microstructural layer beneath the large grains (deeper into the former powder flake).

Further microstructural analysis was performed on the MQIII-F and 19 kW FSPS materials on slices taken parallel to the pressing direction using Transmission Electron Microscopy (TEM) (Figs 8 and 9). Figure 8 shows the TEM images obtained of the MQIII-F material. This reveals the uniform plate-like sub-micron grains in the material; the average size of which is in agreement with that measured from the SEM images. While there is a clear overall crystallographic alignment of the grains it is surprising, given the high remanence of this material, to see that the degree of misalignment is as large as 65° in some cases.

The TEM microstructure observed in the FSPS 19 kW material is markedly different to that of the MQIII-F material (Fig. 9). Here, the large grains present at the former particle surfaces (regions A in Fig. 7) are seen (Fig. 9(a)), with some plate-like growth of smaller grains (regions B in Fig. 7) observed next to this layer (Fig. 9(b)). The residual strain from the rapid deformation process is very clear from the banded contrast seen within the larger grains. At higher magnification (Fig. 9(b)) it can be seen that the grain boundary phase is much thicker and more inhomogeneously distributed in comparison to the MQIII-F material. In addition to this, where the Nd-rich phase usually appears as a clean white phase under TEM, the grain boundary phase in the FSPS material exhibits a speckled texture. This, along with the wavy and diffuse boundaries between the grains and the grain boundary phase, suggests that some dissolved/liquid Nd_2_Fe_14_B became trapped in solution due to the rapid processing and relatively fast cooling rates experienced by the sample. If this is the case, then the magnetic nature of the grain boundary phase may have been altered from an ideal paramagnetic phase (as expected for pure Nd) to a ferromagnetic phase due to the incorporation of Fe. This could therefore represent another explanation for the reduced coercivity in comparison to the MQIII-F material, since neighbouring grains may become magnetically coupled and therefore able to influence one-another’s magnetic polarisation.

Based on the findings of the microstructural investigations it is clear to see that in order to improve the magnetic properties, the microstructure must be homogenised by creating a more even temperature distribution across the powder flakes during sintering. Most importantly, localised overheating at the particle surfaces must be reduced as far as possible in order to curb excessive grain growth and dissolution. This localised overheating occurs at the particle interfaces due to the increased resistivity across the interface; which leads to charge build-up and a greater dissipation of heat in these areas. Hence, by increasing the processing pressure during FSPS the electrical and thermal contact between powder flakes can be improved and it may be possible to avoid local overheating.

Two more samples were therefore prepared using higher applied forces. Both samples were also processed using a lower heating power of 13 kW to avoid excessive overheating. Since the pre-compact can only withstand relatively small forces, one sample was processed using a constant 7 kN force (in place of the original 5 kN force) and the other sample was subjected to a ramped pressure from 5 to 10 kN during the 10 s flash in order to take advantage of the extra strength of the material generated as its density increased. The density of both materials was measured to be near-theoretical.

**Figure 10:**
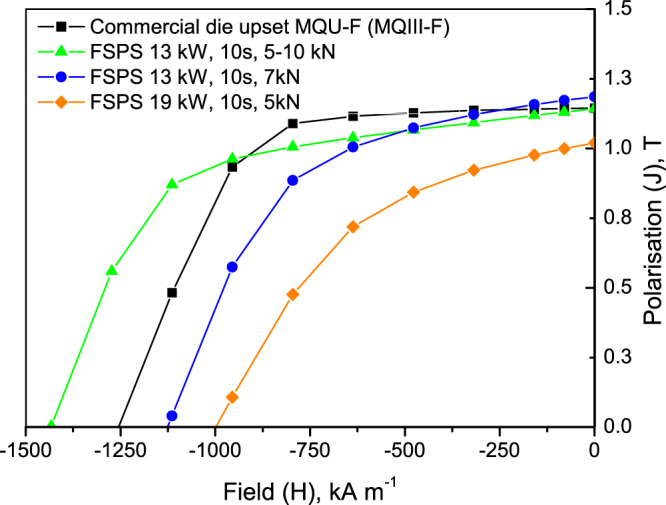
Demagnetisation curves of the FSPS samples processed under lower heating powers and higher applied forces in comparison to the MQIII-F material and the previous ‘best’ FSPS sample discharged under a 19 kW heating power and 5 kN force.

The magnetic properties were measured with a VSM, using the same procedure as employed previously, so as to be directly comparable to the initial ‘best’ FSPS sample and to the MQIII-F material. The measured demagnetisation curves are shown in Fig. 10, alongside the 19 kW initial FSPS sample and the MQIII-F material. It can be seen that the remanence of both materials has improved significantly due to the reduction in heating power and small increase in the applied force; and in both cases is now at least matching the remanence of the MQIII-F material. This indicates that a much greater degree of crystallographic alignment has been achieved in the samples.

Significant increases in coercivity have also been achieved; particularly in the sample that was subjected to the ramped 5–10 kN force, which exhibits a 14% enhancement in coercivity in comparison to the MQIII-F material. This sample in particular therefore exceeds the current state-of-the-art in terms of its magnetic properties.

**Figure 11:**
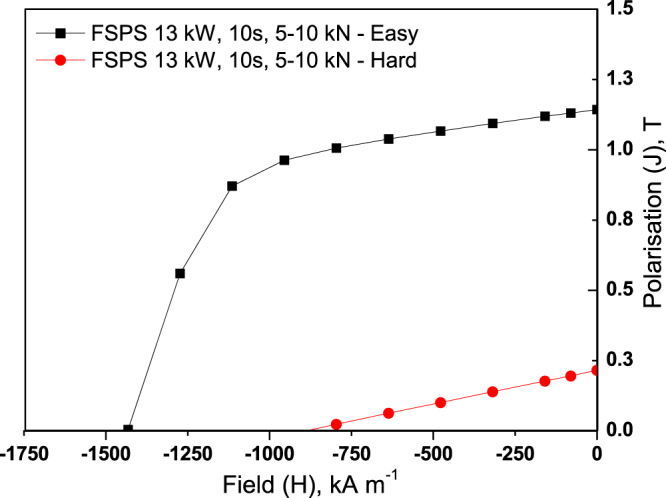
Magnetic anisotropy (demagnetisation curves measured in the ‘easy’ and ‘hard’ directions of magnetisation) of the optimised FSPS sample.

The demagnetisation curves measured in the ‘easy’ and ‘hard’ magnetisation directions (as previously measured) are shown for the ‘optimised’ (ramped 5–10 kN) FSPS sample in Fig. 11. A high degree of magnetic anisotropy is evident and calculated to be 82% (equation (1)).

**Figure 12:**
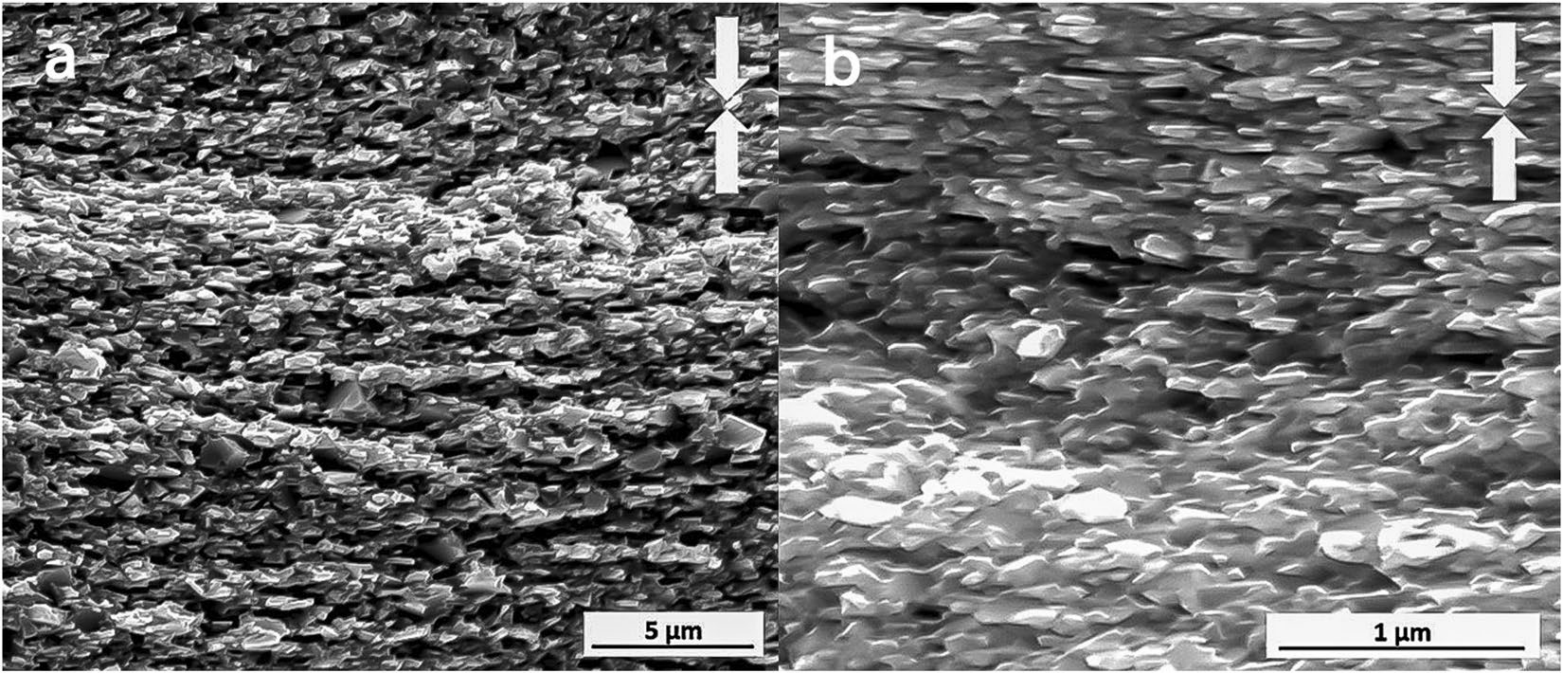
SEM image of the optimised FSPS sample (13 kW, 10 s, 5–10 kN ramp).

**Figure 13:**
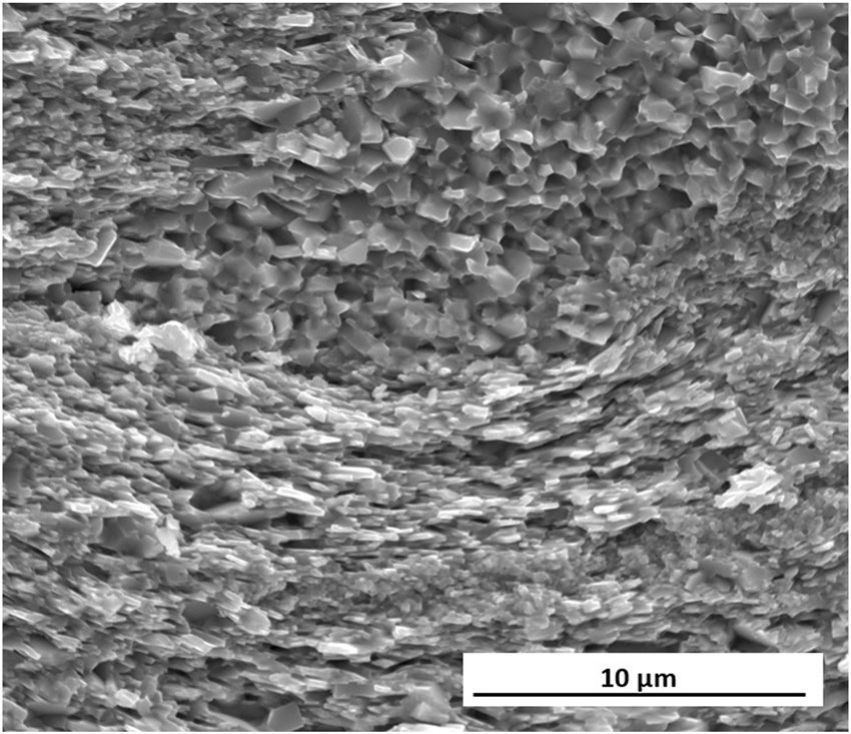
Faceted grain region in optimised FSPS MQU-F.

In order to understand the origin of these marked improvements in magnetic properties and of the enhancement in coercivity in comparison to the MQIII-F material, the microstructure was investigated by SEM imaging of the fracture surface parallel to the pressing direction (Fig. 12). Here it can be seen that a much finer and more homogeneous microstructure has been achieved. Very fine, aligned plate-like grains are observed throughout the sample; the majority of which are no longer than the single-domain grain size (~300 nm). The layered structure observed in the initial FSPS samples is no longer present, with the vast majority of the microstructure consisting of fine, plate-like grains. In some small areas (Fig. 13), some coarser equiaxed grains are still observed; however, even these grains remain sub-micron in size.

The average measured length of the grains in the optimised FSPS material is 272 ± 153 nm, with a width of 61 ± 47 nm and an aspect ratio of 5.2 ± 1.6; hence they are, on average, close to half the size of the grains measured in the MQIII-F material. This can account for the coercivity enhancement measured in the FSPS material in comparison to the MQIII-F material, since a greater volume fraction of grains will be of single-domain size in the optimised FSPS material.

## Conclusions

The novel FSPS processing route for the production of anisotropic, nanograined Nd-Fe-B materials has been applied to a Dy-free melt spun material. Near theoretical densities and high degrees of anisotropy have been achieved. Under low applied forces (5 kN), the electrical and thermal contact between powder particles is poor, and hence there is greater dissipation of heat in these areas, leading to steep temperature gradients through the powder flakes during processing. This leads to the formation of a layered microstructure consisting of coarse grains at the surface of the former flakes, plate-like aligned grains further into the flakes and fine equiaxed grains remaining at the centre of the flakes, where the temperature is coolest. As a result, the magnetic properties of these samples fall short of the benchmark commercial MQIII-F material fabricated from the same melt spun powder using the conventional three step (cold press – hot press – die upset) processing method. By increasing the pressure, and in particular by ramping the force from 5 to 10 kN during the 10 s flash sintering step, the electrical and thermal contact between neighbouring powder flakes is significantly improved. This is evident in the resulting microstructures, which do not exhibit the layered structure and consist mainly of very fine, plate-like aligned grains. The large majority of these grains are finer than the single-domain size, and are nearly half the average size of the grains in the MQIII-F material. This led to an enhancement in coercivity of 14% (to 1438 kA m^−1^) in comparison to the MQIII-F material, which is significant given the Dy-free composition. In addition, the greater volume fraction of aligned grains led to a B_r_ that matches that of the MQIII-F material (1.16 T). This equates to a BH_max_ of 230 kJ m^−3^. This material therefore exceeds the current state-of-the-art and is achieved using a quick and energy efficient process, which could be performed directly to cold-compacts; thereby removing the need for the hot pressing step. With further process optimisation and tooling development/scale-up to industrial level, the FSPS processing route may therefore offer a more economical route to the production of high-performance Dy-free Nd-Fe-B permanent magnets.

## Electronic supplementary material

Supplementary Information
