# Supplementary material for: High coercivity, anisotropic, heavy rare earth-free Nd-Fe-B by Flash Spark Plasma Sintering
Source: Sci Rep. 2017 Sep 11;7:11134. doi: 10.1038/s41598-017-11660-9 (PMC5593872; doi:10.1038/s41598-017-11660-9)
Supplement: Supplementary file 1 — Supplementary Information [file 41598_2017_11660_MOESM1_ESM.pdf]

High coercivity, anisotropic, heavy rare earth-free Nd-Fe-B by Flash Spark Plasma Sintering

Elinor Castle[a*], Richard Sheridan[b], Wei Zhou[b], Salvatore Grasso[a], Allan Walton[b], Mike Reece[a]

a) Queen Mary, University of London, Mile End Road, London, E1 4NS, UK
b) University of Birmingham, Edgbaston, Birmingham, West Midlands, B15 2TT, UK

**Corresponding author: Room 2.08 Nanoforce, Joseph Priestley Building, Queen Mary, University of London, Mile End Road, London, E1 4NS. UK. E-mail: e.g.castle@qmul.ac.uk*

Supplementary Information

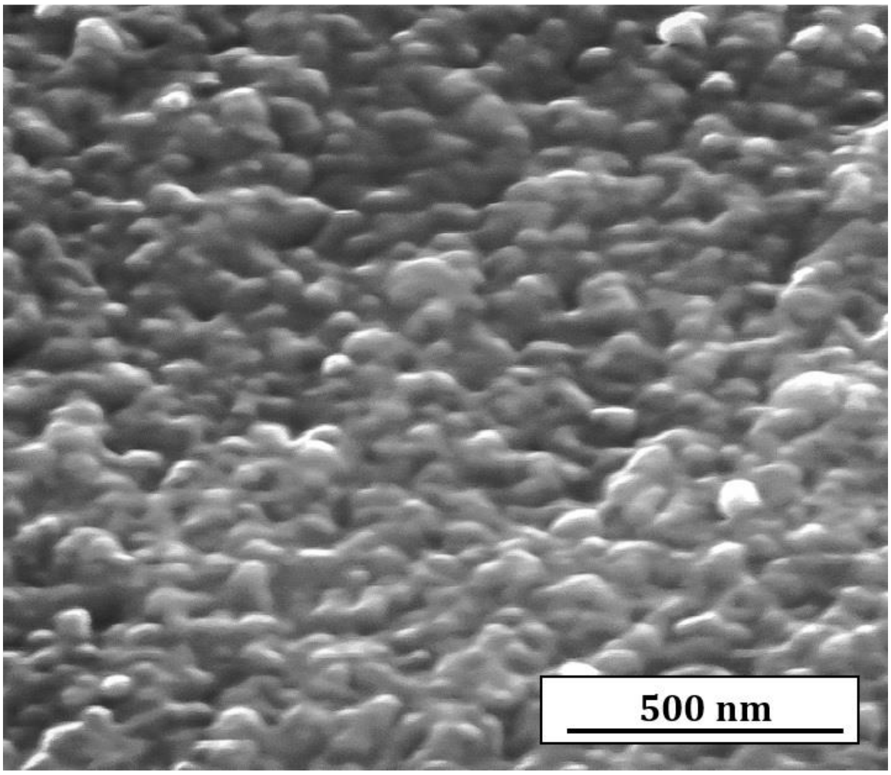

Figure A. *Starting microstructure of the MQU-F powder.*

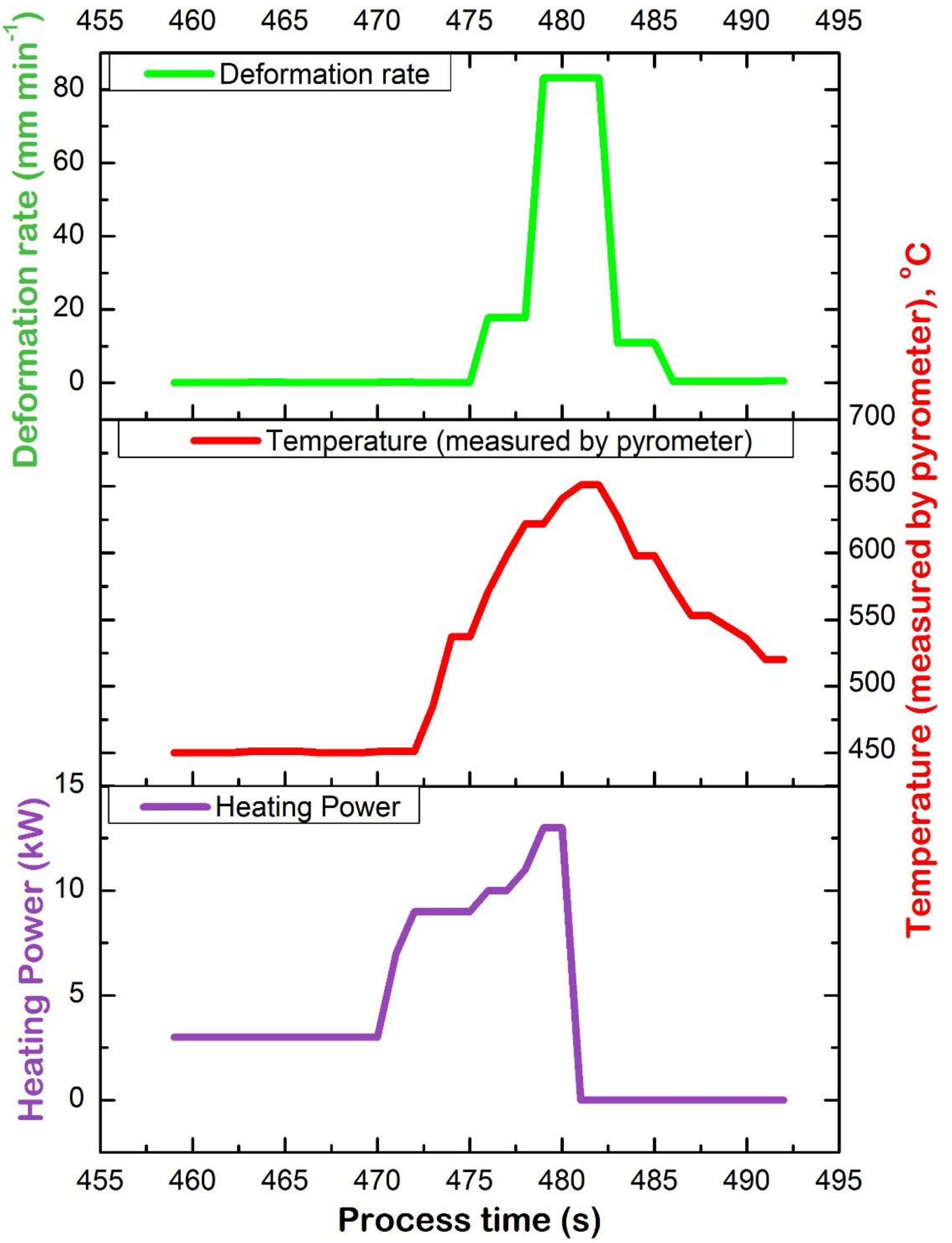

Figure B. *SPS data output for the fully optimised FSPS process. Note that the temperature is recorded by the pyrometer which is focused onto the inside of the top graphite punch, and not directly onto the sample. Actual sample temperatures will be significantly higher than that shown.*
